# Supplementary material for: Causal Relationship between Aging and Anorexia Nervosa: A White-Matter-Microstructure-Mediated Mendelian Randomization Analysis
Source: Biomedicines. 2024 Aug 16;12(8):1874. doi: 10.3390/biomedicines12081874 (PMC11351342; doi:10.3390/biomedicines12081874)
Supplement: Supplementary file 1 [file biomedicines-12-01874-s001.zip › Supplementary Figure/Figure S8/Leaveoneout plot of MR analysis for TL to ubm-b-1674.pdf]

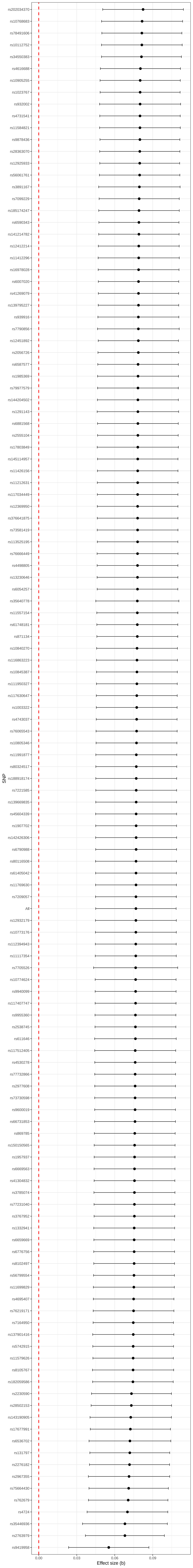
rs202034370
rs10768683
rs78491606
rs10112752
rs34550383
rs4616688
rs10905255
rs1023767
rs932002
rs4731541
rs11584821
rs9878436
rs28363070
rs12925933
rs56061761
rs3891167
rs7099229
rs185174247
rs6590343
rs141214782
rs12412214
rs11412296
rs16978028
rs6007020
rs41269079
rs139795227
rs939916
rs7790856
rs12451892
rs2056726
rs6587577
rs1985369
rs79977579
rs144204502
rs1291143
rs6881568
rs2555104
rs17803849
rs145114957
rs11426156
rs11212631
rs117034449
rs12369950
rs376641875
rs73581419
rs113525195
rs76666449
rs4498805
rs13230646
rs6054257
rs35640778
rs11557154
rs61748181
rs871134
rs10840270
rs116863223
rs10845387
rs111950327
rs117630647
rs1003322
rs4743037
rs76065543
rs10805346
rs11991877
rs80324517
rs188918174
rs7221585
rs139669835
rs45604339
rs1907702
rs142426306
rs6790988
rs80116508
rs61405042
rs11769630
rs7209057
All
rs12932179
rs10773176
rs112394943
rs11117354
rs7705526
rs10774624
rs9940099
rs117407747
rs9955360
rs2538745
rs611646
rs117512405
rs4530278
rs77732866
rs2977608
rs73730598
rs9600019
rs66731853
rs869785
rs150150565
rs1957937
rs6669563
rs41304832
rs3785074
rs77231040
rs3767952
rs1332941
rs6659669
rs6776756
rs8102497
rs56799554
rs11699829
rs4695407
rs76219171
rs7164950
rs137901416
rs5742915
rs11579626
rs8105767
rs182059586
rs2230590
rs28502153
rs143190905
rs17677991
rs6536702
rs131797
rs2276182
rs2967355
rs75664430
rs762679
rs4724
rs35446936
rs2763979
rs9419958
SNP
0.00
0.03
0.06
0.09
Effect size (b)
